# Nicotine replacement therapy for agitation and delirium management in the intensive care unit: a systematic review of the literature

**DOI:** 10.1186/s40560-016-0184-x

**Published:** 2016-11-15

**Authors:** Melanie Kowalski, Andrew A. Udy, Hayden J. McRobbie, Michael J. Dooley

**Affiliations:** 1 Pharmacy Department, Alfred Health, 55 Commercial Road, Melbourne, VIC 3004 Australia; 2 Intensive Care Unit, Alfred Health, Melbourne, Australia; 3 Wolfson Institute of Preventive Medicine, London, UK; 4 Queen Mary University of London, London, UK; 5 Monash University, Melbourne, Australia

**Keywords:** Nicotine replacement therapy, Nicotine withdrawal, Intensive care unit, Critical care, Delirium, Agitation

## Abstract

**Background:**

Active smokers are prevalent within the intensive care setting and place a significant burden on healthcare systems. Nicotine withdrawal due to forced abstinence on admission may contribute to increased agitation and delirium in this patient group. The aim of this systematic review was to determine whether management of nicotine withdrawal, with nicotine replacement therapy (NRT), reduces agitation and delirium in critically ill patients admitted to the intensive care unit (ICU).

**Methods:**

The following sources were used in this review: MEDLINE, EMBASE, and CINAHL Plus databases. Included studies reported delirium or agitation outcomes in current smokers, where NRT was used as management of nicotine withdrawal, in the intensive care setting. Studies were included regardless of design or number of participants. Data were extracted on ICU classification; study design; population baseline characteristics; allocation and dose of NRT; agitation and delirium assessment methods; and the frequency of agitation, delirium, and psychotropic medication use.

**Results:**

Six studies were included. NRT was mostly prescribed for smokers with heavier smoking histories. Three studies reported an association between increased agitation or delirium and NRT use; one study could not find any significant benefit or harm from NRT use; and two described a reduction of symptomatic nicotine withdrawal. A lack of consistent and validated assessment measures, combined with limitations in the quality of reported data, contribute to conflicting results.

**Conclusions:**

Current evidence for the use of NRT in agitation and delirium management in the ICU is inconclusive. An evaluation of risk versus benefit on an individual patient basis should be considered when prescribing NRT. Further studies that consider prognostic balance, adjust for confounders, and employ validated assessment tools are urgently needed.

## Background

Active tobacco smokers are highly represented among critically ill patients, placing a significant additional burden on healthcare systems. Smoking has been demonstrated to have dose-related adverse effects on length of stay and hospital mortality in the critically ill [1]. Moreover, clinical care can often be more complicated due the development of nicotine withdrawal. Symptoms of tobacco withdrawal include irritability, frustration, anger, anxiety, depressed mood, insomnia, and restlessness. Symptoms peak within the first week of smoking cessation and last around 2–4 weeks [2]. It has been proposed that tobacco withdrawal contributes to an increased risk of agitation and delirium in patients admitted to ICU [3, 4], the development of which has been independently associated with inferior clinical outcomes [4].

Delirium is an acute state of confusion. Diagnostic criteria comprise a relatively short onset disturbance in attention and awareness, associated with a fluctuating change in cognition [5]. ICU delirium is common, occurring in 11–80% of critically ill patients [6]. Agitation may exist on its own or in combination with delirium, with a reported frequency of 64% in smokers admitted to the ICU [3]. Development of delirium and/or agitation during admission are linked to adverse events, including a 10% increase in mortality for each additional day spent delirious [7]. Other negative associations include a greater length of time spent mechanically ventilated and in the ICU, increased nosocomial infection, and increased use of psychotropics [8, 9].

Symptoms of tobacco withdrawal have been effectively managed in ward-based and outpatient settings with the use of nicotine replacement therapy (NRT) [10]. NRT primarily acts to reduce the severity of the urge to smoke and other withdrawal symptoms. It is unclear how critically ill patients are affected by these symptoms; hence, there is uncertainty about the benefits of using NRT [11]. A recent systematic review concluded that NRT should only be considered in selected ICU patients, due to a lack of evidence regarding efficacy and safety; however, the primary endpoint of interest was mortality [12]. Rather, the aim of this systematic review was to determine whether management of nicotine withdrawal with NRT reduces agitation and delirium.

## Methods

### Search strategy

A systematic review was conducted of MEDLINE (1946 to July 2016), EMBASE (1974 to July 2016), and CINAHL Plus (1937 to July 2016) using the following terms: nicotine replacement therapy, tobacco use cessation products, smoking cessation, intensive care unit, critical care, nicotine withdrawal, delirium, and agitation. Keywords were combined using Boolean logic. The search was limited to adult human studies written in English. References of retrieved articles were also scanned to identify further studies.

### Study selection

**Fig. 1:**
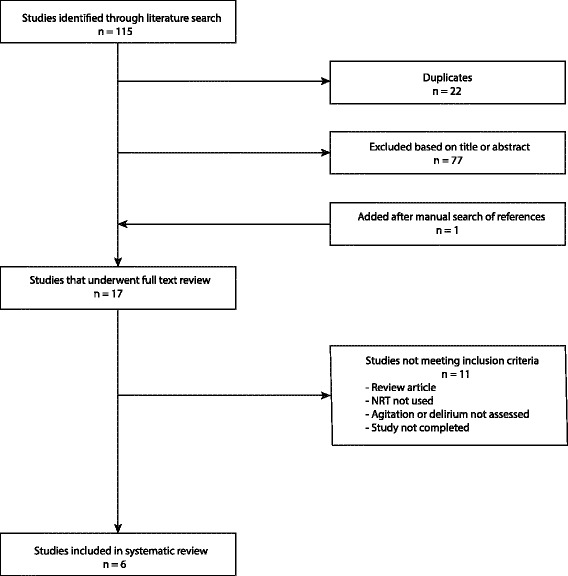
Flow diagram of literature search and study selection. *n* = number of journal articles

Inclusion criteria required a study population of current smokers admitted to the ICU where NRT was used as part of management for nicotine withdrawal. Agitation and delirium was assessed by either quantitative or qualitative measures. Duplicate publications and review articles were excluded. The title or abstract of identified references were examined, and if deemed relevant, full text articles were retrieved and reviewed. A summary of the study selection strategy is illustrated in Fig. 1.

### Data extraction

A full text review was performed to establish if inclusion criteria were met. Data were extracted in a standardised manner. Information included ICU classification, study population baseline characteristics, baseline smoking status, allocation and dose of NRT used, agitation and delirium assessment methods, and frequency of agitation, delirium, and psychotropic medication use.

## Results

The initial search strategy identified 115 citations. A manual search of retrieved references identified one additional study. Duplicates were removed and 77 citations were excluded upon title and abstract review. Full text review was undertaken of the remaining 17 studies to determine eligibility. Eleven studies were excluded (see Fig. 1), leaving six studies eligible for systematic review [13–18]. Study design was of variable quality ranging from a case report [14] to a pilot randomised control trial (RCT) [17]. Study participants were all current smokers admitted either to a medical, surgical, or neurological ICU. Intervention groups, where present, were all prescribed a form of NRT, while studies with control groups received either placebo or no intervention. Mean patient age ranged from 41 to 57.4 years. Details of the included studies are summarised in Table 1.

**Table 1:** Study design and baseline characteristics

First author, year (ref no.)	ICU type	Design, sample size	Mean age (years)	Gender Male %	APACHE II^a^	Mechanical ventilation %	Excessive alcohol intake	Smoking history	Source of smoking history
Mayer, 2001 [13]	Neurological ICU	Case series *n* = 5	52.6	60	–	–	–	“Heavy” tobacco use	Surrogate *n* = 1 Not stated *n* = 4
Honisett, 2001 [14]	Surgical ICU	Case report *n* = 1	41	100	–	100	“Heavy” drinker	“Heavy” smoker	Not stated
Seder, 2010 [15]	Neurological ICU	Retrospective cohort NRT = 128 No NRT = 106	NRT = 50 No NRT = 50	NRT = 34 No NRT = 33	NRT = 11.4 ± 7.4 No NRT = 10.7 ± 7.8	–	NRT = 30% No NRT = 16%	>10 cigarettes/day NRT = 73% No NRT = 47% Pack year history^a^ NRT = 34 ± 29 No NRT = 31 ± 34	Patient/surrogate reporting
Cartin-Ceba, 2011 [16]	Medical ICU	Prospective cohort NRT = 174 No NRT = 156	NRT = 53.8 No NRT = 54.6	NRT = 60.3 No NRT = 53.2	APACHE III NRT = 50 (35–65.5) No NRT = 49 (38–62)	NRT = 69 No NRT = 44	–	Cigarettes/day^b^ NRT = 20 (10–30) No NRT = 15 (10–20) Pack year history^b^ NRT = 30 (18–50) No NRT = 23 (10–45)	Patient/surrogate reporting Tobacco assessment protocol
Gillies, 2012 [18]	Mixed medical/surgical ICU	Retrospective cohort NRT = 73 No NRT = 350	NRT = 55.5 No NRT = 56.3	NRT = 64.9 No NRT = 67.4	NRT = 21.8 ± 15.5 No NRT = 27.2 ± 20.1	–	NRT = 50% No NRT = 21.7% *p* < 0.001	Not reported	Electronic medical records
Pathak, 2013 [17]	Mixed medical/surgical ICU	RCT double-blind pilot study NRT = 20 No NRT = 20	NRT = 57.4 No NRT = 52.3	67.5	NRT = 14.3 ± 9.7 No NRT = 13.8 ± 9.4	NRT = 50 No NRT = 50	–	Packs/day^a^ NRT = 1.2 ± 0.5 No NRT = 1.0 ± 0.4 Years of smoking^a^ NRT = 24.4 ± 10.2 No NRT = 23.3 ± 10.7	Self report At time of written consent

^a^Mean ± standard deviation

^b^Median (interquartile range)

### Assessment of current smoking status

Determination of baseline smoking status varied greatly (see Table 1), with two studies simply reporting all patients as “heavy” smokers [13, 14]. Pack year history could be derived from three studies [15–17]. Two of these studies also reported an average quantity of cigarettes smoked per day [16, 17], while the third study classified patients by those who smoked >10 cigarettes per day or not [15]. One study did not quantify smoking history [18]. The source of smoking history was either self or surrogate reported in three of the six studies [15–17], with one cohort study [16] also utilising a nurse-initiated tobacco assessment protocol. Another study [18] searched for smoking-related documentation via patient electronic medical records. Smoking history sources were not explicitly stated in the case report [14]; a family member reported smoking history for one of the case series [13].

### Allocation of nicotine replacement therapy

The allocation of NRT also varied between studies (Table 1). One study [17] randomised subjects, in a double-blinded manner, to receive either a 21-mg nicotine or placebo patch within 48 h of ICU admission. Another study [16] used a nurse-driven protocol to determine NRT prescribing, with patch doses adjusted for cigarette consumption. Two studies [15, 18] prescribed patients NRT at the clinicians’ discretion. A patch strength of 21 mg was prescribed for all patients in one of these studies [15], while patch strength ranged from 10 to 30 mg/day in the other [18]. Two studies [13, 14] allocated nicotine patches to patients as treatment in response to suspected nicotine withdrawal.

Time to therapy initiation was mostly within 48 h of admission to ICU, although one study [15] included smokers with NRT commenced within 2 weeks of admission but did not specify median time to therapy. In the case series, NRT was commenced within 3–11 days, in response to symptoms of presumed nicotine withdrawal.

### Assessment of agitation or delirium

Agitation and delirium assessment methods ranged from validated tools to subjective description and surrogate markers (see Table 2). One study [16] used the Richmond Agitation-Sedation Scale (RASS) and Confusion Assessment Method for ICU (CAM-ICU) to assess agitation and delirium, respectively, both of which are validated tools with high sensitivity and specificity [19, 20]. The worst daily score for each tool was used to report median RASS score and number of days spent with delirium for each patient throughout the ICU admission. Use of physical restraints was also reported.

**Table 2:** Nicotine replacement therapy, agitation, delirium, and associated risk factors

First author, year (ref no.)	Allocation of NRT	NRT dose and form	Time to NRT therapy (days)	Delirium and agitation assessment method	Incidence of delirium or agitation	Frequency and duration of assessment	New psychotropics prescribed
Mayer, 2001 [13]	Treatment response to suspected nicotine withdrawal	21-mg patch	Range 3–11	Subjective	NRT = 100%	–	Sedation, analgesia, or psychotropic use reported *n* = 4
Honisett, 2001 [14]	Treatment response to suspected nicotine withdrawal	Patch dose not reported	<2	Subjective description of agitation	NRT = 100%	–	Sedation and analgesia *n* = 1
Seder, 2010 [15]	Clinician discretion	21-mg patch	1–14	Delirium definition provided Assessment method not reported	NRT = 19% No NRT = 7%	–	–
Cartin-Ceba, 2011 [16]	Nurse-driven protocol	21-mg patch (14–21 mg)^a^	<1	RASS CAM-ICU Use of physical restraints	RASS^a^ NRT = −1 (−4 to 0) No NRT = 0 (−2 to 0) Positive CAM-ICU days NRT = 23% (169/734) No NRT = 13.1% (75/131) Physical restraint days NRT = 38% (281/734) No NRT = 19.5% (112/573)	Worst daily assessment recorded	Fentanyl equivalence mcg^a^ NRT = 50 (0–874.9) No NRT = 0 (0–472) *P* < 0.001 Lorazepam equivalence mg^a^ NRT = 0.5 (0–11.5) No NRT = 0 (0–2.3) *P* < 0.001 More quetiapine in NRT group More dexmedetomidine and haloperidol in no NRT group
Gillies, 2012 [18]	Clinician discretion	20-mg patch (range 10–30 mg)	2.3 (1.5–5.0)^a^	Validated chart review with prescription of ≥2 anti-agitation drugs as surrogate marker	NRT = 25.7% No NRT = 7.1%	Once per patient	NRT = 25.7% No NRT = 7.1% Required ≥2 anti-agitation drugs *P* < 0.001
Pathak, 2013 [17]	Randomised	NRT = 21-mg patch No NRT = placebo	≤2	Analgesia, sedation, and days on ventilator used as surrogate marker	Mechanical ventilation days^b^ NRT = 1.9 ± 3.7 No NRT = 3.5 ± 5.3	–	Sedation (days) NRT = 1.4 No NRT = 2.7 Analgesia (days) NRT = 1.1 No NRT = 2.1

^a^Median (interquartile range)

^b^Mean ± standard deviation

Another study [18] used a validated chart review to confirm the presence of an acute confusional state along with the prescription of two or more anti-agitation drugs as a marker for agitation or delirium.

The pilot RCT [17] compared use of analgesia, sedation, and days on mechanical ventilation as surrogate markers in order to comment on agitation or delirium. One study [15] provided a definition for delirium but did not describe a method of assessment. The case series and case report provided descriptions of either agitation or delirium but did not report a formal assessment method or frequency.

### Frequency of agitation and delirium

The case report subjectively describes the patient as agitated but does not comment on delirium status. Agitated behaviour is reported as markedly reduced after commencing a nicotine patch [14]. The case series documents five cases of agitated delirium, all of which completely resolve or markedly improve within 24 h of nicotine patch application [13].

One study [15] found delirium to be more prevalent in the group receiving NRT compared to smokers who were not prescribed NRT (NRT = 19% vs no NRT = 7% odds ratio (OR) 3.30; confidence interval (CI) 1.37–7.97; *P* = 0.006). Comparable results were noted in another study [18], with a greater percentage of patients who were prescribed NRT experiencing an episode of agitation or delirium (NRT = 25.7% vs no NRT = 7.1%; *P* < 0.001).

One study [16] found the group prescribed NRT required slightly heavier sedation with a median RASS = −1, compared to a RASS = 0 in the non-NRT group (*P* = 0.02). The percentage of positive CAM-ICU days was also greater for NRT users, with 23% of days spent in ICU with delirium versus 13.1% in those not receiving NRT (*P* < 0.001). Days spent in physical restraints were also significantly greater in the NRT group (NRT = 38% vs no NRT = 19.5%; *P* < 0.001).The pilot RCT [17] noted fewer days spent on mechanical ventilation with nicotine patches (NRT = 1.9 days vs placebo = 3.5 days).

### Psychotropic use

The case report describes post-operative weaning of sedation and patient-controlled morphine analgesia with agitation developing despite adequate analgesia [14]. Repeated doses of sedation, analgesia, and antipsychotics were reported to be required with limited effect in four out of five patients presented in the case series [13].

A cohort study [16] found greater median fentanyl equivalent analgesia use in the NRT group compared to the group without NRT (*P* < 0.001). Benzodiazepine use was also greater in the NRT group (*P* < 0.001). The group without NRT required larger doses of haloperidol and dexmedetomidine.

Another cohort study [18] did not specify quantities of psychotropic medications used, but reported that a higher proportion of NRT patients (26 vs 7% of the no NRT group, *P* < 0.001) required two or more anti-agitation drugs.

The pilot RCT [17] compared days spent in ICU with sedation or analgesia. The mean number of days on sedation was almost half in patients randomised to receive NRT (1.4 vs 2.7 days with placebo). Days receiving analgesia were also less in the NRT group (1.1 vs 2.1 days with placebo). *P* values were non-significant due to inadequate sample size. The investigators comment that the finding of reduced sedation requirements may be linked with reduced agitation.

### Other risk factors for agitation or delirium

Overall reporting of other risk factors for agitation or delirium was poor. Possible confounders include age, severity of illness, and comorbid conditions such as hypertension, alcoholism, and cognitive impairment [4, 6, 9]. Excessive alcohol intake was the most commonly reported additional risk factor for agitation or delirium (Table 2).

One study [15] reported a greater percentage of heavy alcohol use in patients allocated NRT (30%) compared to smokers without NRT (16%). The case report stated the patient was a heavy drinker. The case series considered neurological causes of delirium. Alternative causes of delirium, including illicit drug use, were considered but not alcohol. The remaining studies did not report alcohol consumption or other specific risk factors for agitation or delirium.

## Discussion

Critically ill patients may develop delirium or agitation secondary to a range of causes. As agitation and delirium are associated with numerous adverse effects, it is therefore vital that modifiable risk factors are managed proactively. Nicotine dependence develops through desensitisation and upregulation of nicotinic acetylcholine receptors. This leads to significant changes in dopamine, glutamate, and gamma aminobutyric acid release in active smokers. Abrupt cessation of nicotine inhalation leads to a disruption of this new equilibrium (with previously desensitised receptors becoming unoccupied) and presents clinically as nicotine withdrawal [2, 21]. This systematic review assessed the evidence regarding the use of NRT for nicotine withdrawal within the ICU.

Our key finding is that there is a paucity of high-quality data informing this practice, with one under-powered pilot RCT providing the only interventional evidence. Equally, this review highlights that uncertainty remains regarding whether active smoking is truly a risk factor for ICU delirium, in part due to the deficiencies in identifying active smokers, quantifying baseline smoking status and risk of nicotine withdrawal [22].

The case report and case series describe promising results concerning NRT use in smokers experiencing acute agitated delirium. However, anecdotal reports are at risk of publication bias and should be used to guide clinical decision-making with caution. Further caution should also be applied when validated assessment tools are not used to determine patient outcomes.

Seder et al. [15] found delirium to be more common in the group receiving NRT. However, patients prescribed NRT would likely receive this therapy on the basis of a heavier smoking history. In addition, the method and frequency of cognitive assessment was not reported; hence, it is unclear if NRT was prescribed in response to the onset of agitation or delirium. Neither is it clear if delirium worsened or improved after NRT administration. The group receiving NRT also manifests heavier alcohol consumption. This and other unidentified confounding factors were not adjusted for in analysis; hence, causality cannot be determined.

Gillies et al. [18] have similar interpretive limitations. Specifically, baseline smoking status was not collected, so adjustment for this factor between groups cannot be performed. Robust methods of assessment were absent, meaning active smokers may have been misidentified, and delirium potentially under-reported.

Cartin-Ceba et al. [16] attempted to address these limitations. Allocating NRT using a nicotine dependence assessment protocol allows for appropriate prescribing on the basis of a high likelihood of nicotine withdrawal. This is supported by the observation that the median number of cigarettes smoked per day and years smoked was greater in those prescribed NRT. Of note, increased delirium and use of restraints were identified in the group prescribed NRT. However, baseline differences make causal interpretation difficult, with additional confounding factors, such as heavier sedation requirements, either not reported or not adjusted for. The outcome is further clouded by a trend towards greater antipsychotic requirements in those not prescribed NRT.

The pilot RCT by Pathak et al. [17] is the only publication to date which allows for an unbiased assessment of the effect of NRT in the ICU setting. Reported baseline prognostic factors were balanced due to randomisation. Fewer days requiring sedation, analgesia, and mechanical ventilation in those that received NRT support the hypothesis that this intervention may assist in reducing symptomatic nicotine withdrawal in the ICU, although these findings were not statistically significant due to the small sample size.

Sedatives and analgesics have been shown to increase the risk of delirium [6]. Thus, reducing agitation without having to increase use of sedatives or analgesics is desirable and associated with positive clinical outcomes [23, 24]. Determining whether the trends seen in the pilot RCT [17] also translate to a reduction in agitation or delirium will require a larger study. Assessments should be performed with validated tools rather than use of surrogate markers. An active RCT was identified during the literature search [25]; this may provide further insight into the role of NRT.

Measuring serum nicotine levels achieved with NRT has been validated within hospitalised patients and may support research findings. The critically ill population often have altered pharmacokinetics and augmented transdermal absorption and therefore may experience unexpected serum levels. Accuracy of smoking history assessments is challenging in the ICU population. The Fagerström Test for Nicotine Dependence is a commonly used validated tool, however, only for self-reporting [26]. The nature of ICU admission often deems this impossible and relies on surrogate information. Inaccuracies in this information may alter patient dependency classifications. These factors raise the question of using biochemical markers, such as cotinine, to support information provided from both patients and their families to identify smokers [27].

Overall, this review was not able to determine the true effect of NRT on agitation or delirium in the ICU. Different assessment methods, of varying quality, made interpretation and comparison of agitation and delirium levels difficult. Differences in baseline smoking status between study groups also cloud data interpretation. Reporting and adjusting for confounders was scarce. There is currently insufficient evidence to support prophylactic use of NRT in smokers admitted to the ICU. The decision to prescribe NRT may be considered in patients who are experiencing urges to smoke or who have developed agitation that is attributable to nicotine withdrawal.

## Conclusions

This systematic review was unable to definitively determine the role of NRT in agitation and delirium management in the intensive care setting. Further studies that balance baseline characteristics, adjust for confounders, and employ validated assessment tools are required. In current practice, an evaluation of risk versus benefit on an individual patient basis should be considered when prescribing NRT in the critically ill.

## Abbreviations

NRT: Nicotine replacement therapy
ICU: Intensive care unit
RCT: Randomised control trial
RASS: Richmond Agitation-Sedation Scale
CAM-ICU: Confusion Assessment Method for ICU

## References

[1] Ho KM (2011). Dose-related effect of smoking on mortality in critically ill patients: a multicentre cohort study. Intensive Care Med.

[2] Awissi DK (2013). Alcohol, nicotine, and iatrogenic withdrawals in the ICU. Crit Care Med.

[3] Lucidarme O (2010). Nicotine withdrawal and agitation in ventilated critically ill patients. Crit Care.

[4] Van Rompaey B (2009). Risk factors for delirium in intensive care patients: a prospective cohort study. Crit Care.

[5] American Psychiatric Association, Diagnostic and statistical manual of mental disorders : DSM-5. (Fifth ed.). 2013.

[6] Ouimet S (2007). Incidence, risk factors and consequences of ICU delirium. Intensive Care Med.

[7] Ely E (2004). Delirium as a predictor of mortality in mechanically ventilated patients in the intensive care unit. JAMA.

[8] Jaber S (2005). A prospective study of agitation in a medical-surgical ICU incidence, risk factors, and outcomes. CHEST J.

[9] Dubois MJ (2001). Delirium in an intensive care unit: a study of risk factors. Intensive Care Med.

[10] Rigotti NA, Munafo MR, Stead LF (2008). Smoking cessation interventions for hospitalized smokers: a systematic review. Arch Intern Med.

[11] Afessa B, Keegan M (2010). Critical care support of patients with nicotine addiction. Crit Care.

[12] Wilby KJ, Harder CK (2014). Nicotine replacement therapy in the intensive care unit: a systematic review. J Intensive Care Med.

[13] Mayer SA (2001). Delirium from nicotine withdrawal in neuro-ICU patients. Neurology.

[14] Honisett TD (2001). Nicotine replacement therapy for smokers admitted to intensive care. Intensive Crit Care Nurs.

[15] Seder D (2011). Transdermal nicotine replacement therapy in cigarette smokers with acute subarachnoid hemorrhage. Neurocrit Care.

[16] Cartin-Ceba R (2011). Nicotine replacement therapy in critically ill patients: a prospective observational cohort study. Crit Care Med.

[17] Pathak V (2013). Outcome of nicotine replacement therapy in patients admitted to ICU: a randomized controlled double-blind prospective pilot study. Respir Care.

[18] Gillies MA (2012). Safety of nicotine replacement therapy in critically ill smokers: a retrospective cohort study. Intensive Care Med.

[19] Ely EW (2003). Monitoring sedation status over time in ICU patients: reliability and validity of the Richmond Agitation-Sedation Scale (RASS). JAMA.

[20] Ely EW (2001). Evaluation of delirium in critically ill patients: validation of the Confusion Assessment Method for the Intensive Care Unit (CAM-ICU). Crit Care Med.

[21] Paolini M, De Biasi M (2011). Mechanistic insights into nicotine withdrawal. Biochem Pharmacol.

[22] Hsieh SJ (2013). Cigarette smoking as a risk factor for delirium in hospitalized and intensive care unit patients. Ann Am Thorac Soc.

[23] Pandharipande P, Ely EW. Sedative and analgesic medications: risk factors for delirium and sleep disturbances in the critically ill. Critical Care Clinics. 2006;22(2): p. 313-27, vii. 10.1016/j.ccc.2006.02.010 16678002

[24] Woods JC (2004). Severe agitation among ventilated medical intensive care unit patients: frequency, characteristics and outcomes. Intensive Care Med.

[25] Wageningen GVH. Nicotine replacement therapy in the intensive care unit. http://clinicaltrials.gov/show/NCT01362959. Accessed 25 Aug 2016.

[26] Heatherton TF (1991). The Fagerström test for nicotine dependence: a revision of the Fagerstrom Tolerance Questionnaire. Br J Addict.

[27] Hsieh SJ (2011). Biomarkers increase detection of active smoking and secondhand smoke exposure in critically ill patients. Crit Care Med.

